# Dietary Pattern and Its Correlates among Lithuanian Young Adults: Mediterranean Diet Approach

**DOI:** 10.3390/nu12072025

**Published:** 2020-07-08

**Authors:** Brigita Mieziene, Arunas Emeljanovas, Natalja Fatkulina, Rimantas Stukas

**Affiliations:** Institute of Health Sciences, Vilnius University, 03101 Vilnius, Lithuania; arunas.emeljanovas@mf.vu.lt (A.E.); natalja.fatkulina@mf.vu.lt (N.F.); rimantas.stukas@mf.vu.lt (R.S.)

**Keywords:** eating habits, non-Mediterranean country, young people, physical activity, cross-sectional study

## Abstract

A poor diet leads to serious health risks and accounts for a significant proportion of deaths. Young adults are the population whose health behaviors particularly need to be studied in terms of nutrition because they are in a period of life when the effects of nutrition accumulate to health outcomes that usually appear later in life in forms of disease or infirmity. The aim of this study is to examine the dietary pattern and its relationships with physical activity and covariates, such as body mass index, gender, age and education among Lithuanian young adults of 18–36 years old. A cross-sectional study was performed among Lithuanian young people aged 18–36 years. Snowball sampling—a non-probability, convenient sampling strategy—was used. In total, data were collected from 3031 study participants: 1723 (56.8%) were male and 1308 (43.2%) were female. The mean age was 23.72 ± 4.80 years. Adherence to a dietary pattern was evaluated using the Mediterranean Diet Adherence Screener (MEDAS). Physical activity, height, weight and education data were also gathered. About 7% of young people fully complied with the health-related Mediterranean diet, and one-third had poor compliance. Non-compliance is mostly related to the underconsumption of olive oil, nuts, fish, seafood, legumes and wine, as well as the overconsumption of red meat. The female gender (β = 0.26; *p* < 0.01), higher education (β = 0.30; *p* < 0.01) and a sufficient level of physical activity (β = 0.15; *p* < 0.01) predict a healthier diet. These study results should be considered by nutrition policymakers and public health authorities to improve policies and develop intervention plans for improving the nutrition habits of young adults in order to prevent health-damaging outcomes later in their life.

## 1. Introduction

A poor diet leads to serious health risks such as heart disease, certain types of cancer [1] and type 2 diabetes [2]. Consequently, poor diet accounts for 18% of deaths in the European Union [3].

The healthy diet proposed by the World Health Organization (WHO) is similar to the Mediterranean dietary pattern [4]. The beneficial effects of the Mediterranean diet (MD) have been confirmed in many studies. A recent meta-analysis showed a beneficial role of the MD in cardiovascular disease, with a decreased risk of about 20–25% for those with the highest adherence compared with those with the lowest compliance [5,6]. Greater adherence to a Mediterranean diet is associated with significant cardiovascular health improvement [7], even in non-Mediterranean populations [8]. A review of the scientific literature also identified that the MD is associated with a reduction in overall cancer rates, as well as significantly lower rates of digestive tract cancers [9]. The authors of a meta-analysis concluded that adherence to the MD may contribute to the prevention of a series of brain diseases [10], specifically a lowered incidence of Parkinson’s disease and Alzheimer’s disease [11,12]. The MD is also related to a significant reduction in overall mortality, mortality from cardiovascular diseases and the incidence of, or mortality from, cancer [11,12]. Evidence of these effects was found even within the same non-Mediterranean country; for example, in Australia, where the choice of food is the same for everyone, Anglo-Celts had a 33% higher mortality risk than Greek-Australians, depending on diet alone [13].

In general, the MD is a scientific concept of a cultural model of healthy eating in areas where olive oil tree growth prevails [14,15]. Besides olive oil, the MD is dominated by a high consumption of plant-based foods such as vegetables, legumes and fruits. Antioxidants are a common element in these foods and provide an explanation of their health benefits. Green vegetables in the form of salads and pies contain very high quantities of flavonoids [16], which link this diet to health. The MD also involves the regular, moderate consumption of wine [17]. Results of a recent meta-analysis suggest that adherence to the MD coupled with physical activity, both behaviors that reflect the Mediterranean lifestyle [18], have even more beneficial effects on metabolic syndrome [19].

However, it is argued that the MD is no longer Mediterranean because it has a number of nutritional characteristics that are different from the traditional Mediterranean diet [19]. Because of globalization, in the context of changing patterns of nutrition, cultural traditions of eating have changed in Mediterranean countries as well [19]. In particular, these changes are related to the young part of the population, as only 8.5–40% of adolescents in Mediterranean countries comply with MD recommendations [20,21,22]. Moreover, there is evidence of greater adherence to the MD in non-Mediterranean countries in comparison with Mediterranean countries [23,24]. On the other hand, the availability of traditional Mediterranean products such as olive oil, fruits, fish and seafood in non-Mediterranean European countries is also growing [25]. Therefore, the effect of the traditional MD could be transferable to non-Mediterranean populations.

A population-based study demonstrated some positive changes in the eating habits of the Lithuanian adult population during the post-communist transition period (since 1990). There was an increase in the use of vegetable oil in cooking and in the frequency of consumption of fresh vegetables. Conversely, the use of butter on bread decreased, and the proportion of women drinking high-fat milk declined [26]. However, there are no studies examining the eating patterns of adults in terms of the MD. A study in Lithuanian adolescents, who were already born when market globalization was expanding, showed unsatisfactory results, as only 14% of them fully complied with the MD versus 20% who poorly complied. In particular, the study identified insufficient consumption of vegetables, pulses, fish and nuts [27]. Meanwhile, young adults are the population whose health behaviors particularly need to be studied for two main reasons. First, the ages between 18 and 36 constitute a period of life when health behaviors accumulate their effects on health outcomes that usually appear later in life in forms of disease or infirmity. Second, it is likely that at this age, when young adults begin their lives independently from their parents, that their eating habits also go through changes or reshaping and may dramatically differ from the eating patterns of adolescents who live with their parents.

The current study follows the recent trends in nutritional research beyond the classical analytical approach of only assessing exposure to single nutrients or isolated food items: instead, this study focuses on overall dietary patterns [5] because, among other advantages, the compensatory effect of consumption of separate food items is considered.

Therefore, the aim of this study is to examine the dietary pattern and its relationships with physical activity and covariates, such as body mass index, gender, age and education among Lithuanian young adults of 18–36 years old.

## 2. Materials and Methods

### 2.1. Study Design

A cross-sectional study was performed among Lithuanian young people aged 18–36 years. Snowball sampling—a non-probability, convenient sampling strategy—was used. An initial group of individuals was selected from available participants (university, college students, members of youth organizations, professional societies, members of gym clubs). Then, these participants were asked to indicate other potential members to enroll them in the study in an effort to represent the various strata of the 18–36-year-old sample population according to the following criteria: gender and education. An online questionnaire was shared through popular social networks and emails to personal and professional contacts. The study procedure took approximately 15 min.

### 2.2. Participants

In total, data were collected from 3031 study participants: 1723 (56.8%) were male and 1308 (43.2%) were female. The mean age was 23.72 ± 4.80 years. All participants were informed about the goals of the study as well as the anonymity of their participation, and respondents agreed to participate in the study by submitting their online questionnaire. The study was conducted in accordance with the Declaration of Helsinki, and the protocol was approved by the Vilnius Regional Biomedical Research Ethics Committee (No. 2019/2-1099-597).

### 2.3. Measurements

#### 2.3.1. Dietary Pattern

A healthy dietary pattern was defined as a diet based on whole or minimally processed foods and foods that included health-protective ingredients and lacked unhealthy items, such as fast food and sugar-sweetened beverages. Adherence to a dietary pattern was evaluated using the Mediterranean Diet Adherence Screener (MEDAS) [28], which was previously validated in Spanish and German adult populations [28,29]. This tool was previously used to evaluate the dietary habits of Lithuanian conscripts [30]. MEDAS includes 14 items: 2 indicate food-intake habits such as the use of olive oil and the preference for white versus red meat, and the other 12 items capture the frequency of consumption of olive oil, animal fat, vegetables and fruits, fish, nuts, commercial pastries, sugar-sweetened beverages and dishes with homemade sauce. The questionnaire uses pre-defined goals for the consumption of specific food items [31]. In accordance with these goals, each item was scored as 0 (does not meet the healthy eating criteria) or 1 (meets the healthy eating criteria). The total score was calculated by summing all item scores. The MEDAS score was classified into three categories: ≤7 indicated low adherence, 8–9 indicated medium adherence and ≥10 indicated high adherence to the Mediterranean diet [28].

#### 2.3.2. Physical Activity

The pyramid of the MD also includes physical activity at its base [32]. The evaluation of moderate-to-vigorous physical activity (MVPA) in this study was based on the WHO definition: moderate physical activity noticeably accelerates the heart rate and includes activities equivalent in intensity to brisk walking or bicycling; vigorous physical activity causes rapid breathing and substantially increases heart rate, and it includes activities such as jogging, aerobic dancing and bicycling uphill [33]. MVPA in this study was quantified by identifying how many hours and minutes a person is physically active in a week. In two open questions, the study participants were asked to identify (1) how often and (2) how long they participated in the listed physical activities or activities of similar intensity within the last 7 days. Detailed descriptions and examples of what constitutes moderate and vigorous physical activity were presented. In their answers, participants identified the exact numbers of times/week and hours and/or minutes per bout. The number of minutes spent in MVPA was totaled. The threshold of 150 min, in accordance with WHO recommendations, was used to distinguish participants into those not meeting health-related physical activity requirements (<150 min/week) and those meeting health-related physical activity requirements (MVPA for ≥150 min/week) [34].

#### 2.3.3. Covariates

Height and weight were self-reported, and body mass index (BMI) was calculated. Participants were divided into not-overweight (BMI ≤ 24.99) and overweight or obese (BMI > 25) groups according to overweight and obesity thresholds. Education was indicated by asking the highest education level achieved by the participants at the time of the study. Participants were allocated into two groups: (1) those with a university or college degree and (2) those with less than a university/college degree. Gender was also considered as a covariate.

### 2.4. Statistical Analysis

Data were analyzed using SPSS 24.0 software (SPSS Inc., Chicago, IL, USA). Descriptive statistics were employed to determine the means and percentage distributions of variables used in the study. The chi-square test was used to identify relationships between nominal and categorical study variables. The Fisher exact probability test for a two-row by three-column contingency table was applied in appropriate cases. The relationships between dietary patterns and physical activity were identified using hierarchical linear regression analysis, controlling for the covariates gender, BMI and current education. Skewness and kurtosis of standardized residuals in the regression analysis were in the range between −1 and 1. A comparison of the mean difference was performed using Student t criteria. For the independent samples *t*-test, Cohen’s d was calculated to evaluate the effect size of the mean difference. Statistical significance was set at a *p*-value of less than 0.05.

STROBE (STrengthening the Reporting of OBservational studies in Epidemiology) Statement—checklist guidelines were followed in organizing this paper.

## 3. Results

Descriptive statistics of the study sample are summarized in Table 1 and indicate that only about 7% of Lithuanian 18–36-year-old young people fully comply with the health-related Mediterranean diet. Among all young males, just 2% fully comply, in contrast to almost 14% of females, revealing a 12% difference (*p* < 0.05) between genders. Meanwhile, a quarter of all young people have poor adherence to the MD. Within gender groups, about half (45%) of young male adults have poor adherence; however, among females, one out of five (20.4%) have poor adherence, which is almost a 25% difference (*p* < 0.05) between genders. Overweight or obesity is found in a quarter of young Lithuanian adults. Within gender groups, one-third (33.4%) of males are either overweight or obese in comparison with one out of seven females (14.4%; *p* < 0.05). About 40% of the young people in this study completed higher education, with one-third (31.2%) of all males and almost half (49.7%) of females with a university or college degree. As many as two out of three (64%) Lithuanian young people achieve the minimal requirements of 150 MVPA min/week for physical activity: 65.4% of young males are sufficiently physically active, which is almost 4% more than females (61.5%; *p* < 0.05).

Hierarchical linear regression was employed to identify the predictive value of physical activity for the MD, which was put into the equation as a continuous variable of the total MEDAS score, controlling for BMI, gender, age and education (Table 2). The binarized BMI included in Model 1 is revealed to be a significant covariate (β = −0.08, *p* < 0.05), and overweight or obese young people are found to have a poorer diet than individuals who are not overweight. However, BMI covers just 0.5% of MD variance. The results of Model 2, which includes sociodemographic variables, reveal that the female gender (β = 0.26; *p* < 0.01) and higher education (β = 0.30; *p* < 0.01), each independent of the other, are predictors of better adherence to the MD. Age is not related to the MD in Model 2. Sociodemographic variables cover 19% of the MD variance. The results of Model 3 indicate that a sufficient level of physical activity, after controlling for covariates in Model 1 and Model 2, predicts a healthier diet (β = 0.15; *p* < 0.01). Those young people aged 18–36 years old who accumulate at least 150 min/week of MVPA tend to maintain a healthier diet. Physical activity covers 2% of the MD variance.

The general overview of compliance with the MD among young Lithuanians reveals that there are significant total mean differences between gender, education and physical activity groups, with those who are female, have a college or university degree and are sufficiently physically active adopting healthier dietary patterns in general (Table 3). Lithuanian young adults meet the recommendation for the minimum number of fruits consumed per day (3.56 ± 2.8). Sufficient physical activity and education at less than a college degree are associated with the consumption of more fruits than insufficient activity and a university or college degree (*p* < 0.05). Lithuanian young adults also meet the recommendation to eat two or more dishes with a homemade sauce (3.08 ± 2.5). Males and those with a university or college degree eat dishes with a homemade sauce more often than females and those with less education (*p* < 0.05). Among all participants, consumption of butter, margarine or cream is less than one recommended serving per day (0.96 ± 1.2), and males and those with less than a college education consume more than females and those who are more educated (*p* < 0.05). Young Lithuanians drink less than one recommended sweet and carbonated beverage per day (0.47 ± 0.8), and those who are overweight/obese, male, less educated and not sufficiently physically active drink more sweet beverages than those who are not overweight, female, more educated and sufficiently physically active (*p* < 0.05). The recommendation to consume commercially baked sweets or pastries fewer than three times per week is also met (1.90 ± 1.8) in the general population and differs only among education groups: those with less than a college degree consume more non-homemade pastries than those who are more educated (*p* < 0.05). The consumption of vegetables approaches the recommended norm of two servings or 400 g per day (1.75 ± 1.5), and those who are not overweight, female, more educated and sufficiently physically active consume more vegetables than those who are overweight or obese, male, less educated and not sufficiently physically active (*p* < 0.05). However, only 19.3% of young Lithuanians use olive oil as their main culinary fat, with a mean of 2.62 ± 2.6 tablespoons per day instead of the recommended 4 tablespoons. The consumption of olive oil is found more often among females and those with a university or college degree than among males or less-educated individuals. Sufficiently physically active individuals consume more olive oil than those who are not sufficiently physically active. Consumption of legumes and fish or seafood is far from the recommended three servings per week (1.13 ± 1.4 and 1.20 ± 1.3). Those who are not overweight, more educated and sufficiently physically active consume more legumes than those who are overweight or obese, less educated and not sufficiently physically active (*p* < 0.05). Females, those with a university or college education and those who are sufficiently physically active consume more fish or seafood than males, those who are less educated and those who are not sufficiently physically active (*p* < 0.05). The recommendation for consumption of red meat is not followed in the study population, who consume almost twice as much (1.85 ± 1.2) as the recommended amount. Males, those with less than a college education and those who are sufficiently physically active consume more red meat than that consumed by females, those who are more educated and those who are not sufficiently physically active (*p* < 0.05). However, there is a preference for white meat to red meat in 69% of the population of 18–36-year-old young people. Consumption of white meat is more frequent among those who are not overweight, female, more educated and sufficiently physically active than their overweight or obese, male, less educated and insufficiently physically active counterparts (*p* < 0.05). Instead of the recommended three servings of nuts per week, Lithuanian young adults consume about two servings (1.98 ± 2.8), and those who are overweight or obese, more educated and sufficiently physically active consume more nuts than those who are not overweight, less educated and not sufficiently physically active (*p* < 0.05). The consumption of wine differs remarkably from the recommendation, with an average of just about half of a glass per week (0.46 ± 1.2) instead of seven. Those who are not overweight, female and are more educated drink more wine than those who are overweight or obese, male and are less educated. The effect sizes of all the mean comparisons in Table 3 are presented in Tables S1–S4 in Supplementary Materials. 

## 4. Discussion

This study aimed to analyze the dietary patterns of Lithuanian young adults aged 18–36 years old in terms of the Mediterranean diet and identify differences among individuals who are sufficiently physically active and not active and those who are overweight and not overweight, as well as relationships with the main sociodemographic characteristics, such as gender, age and education.

### 4.1. Pattern of Mediterranean Diet

The results of the current study raise concerns that Lithuanian young adults are at risk of developing diet-related diseases and succumbing to premature death, as only about 7% of them fully comply with an MD health-related type of diet, two-thirds have average compliance and around one-third show poor compliance. The recent statistics within the general population of Lithuanians show that around one-third of them die from illnesses caused by poor nutrition, and the rate is 12% higher than that in other EU countries. Among those illnesses, cardiovascular diseases are the leading cause of death in Lithuania, with the highest mortality rate from ischemic heart disease among countries in the EU [3]. Other studies have shown that individuals who are more adherent to the Mediterranean diet are less likely to develop cardiovascular risk factors [35]. Similar benefits of Mediterranean-type dietary patterns on cardiovascular disease risk have been found even in non-Mediterranean populations [5]. Furthermore, a literature review revealed that the MD is associated with a reduction in overall cancer rates, which is the second major cause of death in the country, as well as significantly lower rates of digestive tract cancers [9]. For instance, in the US, which also has high rates of diet-related diseases, among a similarly aged population, only 7% had good adherence to the MD [36]. In comparison, good adherence is found in 24% of Spanish university students [37].

The poor diet of Lithuanian young adults is especially related to the underconsumption of olive oil, which is the key food item in the MD and is recommended to be consumed with every meal [38]. Only 19.3% of young Lithuanians choose olive oil as the main source of fat, underusing it in terms of the amount as well. Nevertheless, up to 50% of people in other non-Mediterranean countries use olive oil as their main fat for cooking [39]. However, the consumption of animal fats, such as butter or cream, is not high either and does not exceed the recommendations to consume less than 12 g/day. Research suggests that cardiovascular diseases and some cancers (colorectal, breast and prostate cancer) are related not to the amount but to the type of fat, with olive oil being beneficial for health [16].

The consumption of vegetables among the Lithuanian population of young adults almost meets the recommended norm. Moreover, young Lithuanians meet the recommendation of fruit consumption. However, there is a remarkable underconsumption of legumes and fish or seafood. Other studies and meta-analytic evidence show that those consuming more vegetables as well as fruits, nuts, legumes, fish and meat/poultry and moderate amounts of wine have better cardiovascular and cognitive health than those consuming less [12,39]. Another meta-analysis specified that even low (1 serving/wk) and moderate (2–4 servings/wk) consumption of fish intake might reduce the risk of coronary heart disease mortality [40]. The beneficial effects of fish on cardiovascular health are attributed to long-chain n–3 PUFAs, protein, vitamin D and selenium, and they have interaction effects on cardiovascular outcomes [41].

The discussion of the different quality and nutritional content of food items in Mediterranean and non-Mediterranean countries should also be mentioned. For instance, it is argued that the nutritional content of fish is determined by the type of fish, the diet of the fish and the method of preparation, which can be totally different among Mediterranean and non-Mediterranean countries [19]. Mediterranean and non-Mediterranean countries vary by their preference of different kinds of vegetables: while Mediterranean youth choose tomatoes, cucumber, cabbage, radishes, spinach and lettuce, other young Europeans prefer “Brussels” sprouts, green peas and carrots [19].

Drinking wine in Lithuania is not found to occur in high amounts; it is drunk more by specific subpopulations, such as women and people with high education. On average, Lithuanians drink just about half of a glass per week instead of at least seven as recommended. By following this recommendation, the risk of cardiovascular disease could be reduced because of the phenolic compounds found in wine, which are beneficial for health [19]. Nevertheless, in general, alcohol consumption is a major behavioral health problem in Lithuania, with 12.3 L of alcohol consumed per adult in 2017, which makes Lithuanians the heaviest drinkers in the EU, exceeding the EU alcohol consumption average by 25% [3]. Instead, beer and spirits have been long considered the traditional alcoholic drinks. In Mediterranean countries, wine is a typical alcoholic drink and is drunk with a meal, which slows the rate of absorption of alcohol from the gut. In contrast, beer and spirits, popular in Lithuania and some other non-Mediterranean countries, raise the absorption rates and may result in the production of carcinogenic metabolites, especially when drunk on an empty stomach [19].

In the current study, people aged 18–36 years old favor red meat more than is considered healthy, as they consume almost twice as much red meat than is recommended. However, 69% of these young people prefer white meat to red meat. A meta-analysis concluded that red meat consumption is associated with a small increase in the risk of colorectal cancer [42]. The negative effects could be increased even more when cooking meat at high temperatures and when using marinades that do not contain virgin olive oil, onions, garlic, herbs or red wine, which are high in antioxidants [19]. Moreover, it is argued that the fatty acid profile of Mediterranean animals is healthier than that of non-Mediterranean animals [19]. This makes red meat consumption in non-Mediterranean countries even more health-damaging.

### 4.2. Association of Mediterranean Diet with Physical Activity

An adoption of MD, along with physical activity, significantly reduces the coronary risk and prevents acute cardiovascular diseases [43]. Among Lithuanian young adults aged 18–36 years old, 64% achieve the minimal requirements of 150 MVPA min/week for physical activity. Results of the current study indicate that a sufficient level of physical activity, after controlling for gender, age, BMI and education, still predicts a healthier diet. Similar results were obtained in a sample of 14–18-year-old adolescents: more physically active Lithuanian high school students were more adherent to the MD [27], but in 18–26-year-old Lithuanian male conscripts, a relationship between MVPA and the MD was not found [30]. The findings of other studies in Sicily [44,45], Spain [46,47] and Iran [48] are in line with our results, showing that more physically active adults adopt a healthier diet.

This study’s results show that those who are sufficiently physically active consume a higher amount of olive oil than that consumed by insufficiently active individuals. The results of one clinical trial revealed that replacing typical Western types of fats with olive oil was associated with increased daily physical activity [49].

In this study, differences were also observed in the consumption of vegetables and fruits: sufficiently physically active participants consume higher amounts of these foods than those who are not sufficiently physically active. Studies in the field of sports nutrition have stated that there is a relationship between lifetime ultra-endurance exercise and lifetime vegetable and fruit consumption. Meanwhile, ultra-endurance exercise was negatively related to the Western type of dietary pattern in the 19–30-year age group and positively related to the fruit and vegetable dietary pattern in the 31–45-year age group [50].

In the current study, sufficiently physically active young adults also consume greater amounts of fish and seafood and more often prefer white meat to red meat compared with those who do not achieve a sufficient level of physical activity. Another randomized control study found that an experimental group receiving fatty fish three times per week had a regular pattern of physical activity throughout the intervention period, while a control group receiving an alternative meal (e.g., chicken, pork and beef) with the same nutritional value showed a more irregular pattern of physical activity and a significant reduction in physical activity over time [51]. On the other hand, physical activity, especially vigorous activity, lowers the risk of gastrointestinal disorders caused by the consumption of red meat [52]. Therefore, physical activity also has a protective effect against a health-damaging diet.

The results of a cross-sectional survey in a large sample of adolescents in the US indicated that the consumption of soda and sugar-sweetened beverages was associated with sedentary behaviors and a number of other unhealthy dietary practices [53]. The results of this study are in line with these findings, which indicate that young people who are not sufficiently physically active more often consume sugar-sweetened or carbonated beverages than their sufficiently physically active counterparts.

### 4.3. Covariates

Females demonstrate significantly higher adherence to the MD than males at a clinically significant level of more than 1 point of the MEDAS score. They also choose olive oil twice as often as males. Furthermore, compared with males, females eat more vegetables and fish and more frequently prefer white meat to red meat. Males consume more red meat, hamburgers or processed meat, non-healthy fats, and more sweetened or carbonated beverages than females. Results of studies on the role of gender in compliance with the MD have been inconsistent: greater compliance was found in females in the US [36] and Sicily [54], whereas more compliance was found in males in Iran [48] and among Spanish students [37].

Those having a university/college degree more often come close to meeting the guidelines of the MD. This result is in line with other studies showing that more educated people adopt healthier diets [54,55]. This could be premised on more educated individuals having more nutritional knowledge and being aware of the pros of a healthy diet and cons of an unhealthy diet, thus continuing to follow healthy nutrition guidelines irrespective of the higher availability of unhealthy foods [45].

In the current study, young people who are overweight or obese have a poorer diet than those who are not overweight. Specifically, the latter consume more vegetables and legumes, drink more wine and more frequently prefer white meat to red meat than overweight or obese young people. In contrast, overweight or obese young people consume more sweetened or carbonated beverages, but they also eat more nuts than those who are not overweight. These results are consistent with other studies showing that people who had a healthy BMI were more likely to have higher adherence levels to the MD, which was associated with lower diastolic blood pressure [55]. Moreover, higher adherence to the MD was inversely related to the risk of weight gain over five years in male adults [56] and positively related to weight loss in overweight adults [57]. There were no associations found between the consumption of olive oil and BMI. Nevertheless, the MD has been considered a high-fat diet, but compliance to this dietary pattern is related to favorable effects on weight maintenance, probably because the high content of monounsaturated fatty acids is related to the regulation of insulin in the blood and thus prevents the development of overweight and obesity [58].

In summary, what makes the diet of young Lithuanian adults poor is the underconsumption of olive oil, nuts, fish, seafood, legumes and wine, as well as the overconsumption of red meat. The risks associated with having a poor diet are greater for overweight or obese individuals, males, those with less than a college education and those who are not sufficiently physically active.

National health policies should promote collaboration and cooperation that foster a healthy diet within and between different institutions, starting from school, college, university, workplaces and primary health care units in order to assist young people in developing healthy eating habits. Health education, in addition to other health behaviors, should include awareness-raising promotional activities to tackle the unhealthy or lack of healthy eating habits, specifically the consumption of olive oil, nuts, fish, seafood and legumes and the avoidance of red meat, as well as the consumption of fruits and vegetables. Additionally, behavioral changes could be induced and supported through social capital interventions and social marketing directed to promote a healthy diet along with physical activity as a personal value, especially in an attempt to reach those who are already overweight or obese, less educated and male.

### 4.4. Strengths and Limitations

First, to the best of our knowledge, this study is the first to assess MD adherence in a young population of 18–36-year-olds in Eastern, Central and Northern Europe in general and in the Baltic States in particular. As Lithuania, Latvia and Estonia share the same geographical region and socioeconomic situation, this study’s results can reflect the region in general. Second, besides nutrition, another important part of the Mediterranean lifestyle—physical activity—was measured. However, measures were self-reported, which might lead to inaccurate physical activity data, and either over- or underestimation. On the other hand, a large study sample size reduces the probability of false findings [59]. Third, the results are based on data collected from a big sample of participants; although it was drawn using a non-probability sampling technique, it still represents the general proportions of education groups in the country. Among the limitations, the first is that this study is an observational trial only, and it is difficult to determine the cause and effect. Second, even though a representative number of participants was included in the study, it is still the result of the non-probability sampling technique. Third, although the MEDAS is a validated tool, people tend to overestimate their healthy eating on the MEDAS when compared with their actual food intake [60]. However, it is still agreed that the MEDAS provides reasonable estimates to assign the same absolute MD score ratings as food records [60]. Finally, the MEDAS is not specifically validated among the Lithuanian population. However, the potential of the MEDAS to assess adherence to the MD is similar in both Mediterranean and non-Mediterranean countries [60].

## 5. Conclusions

This study showed that people aged 18–36 are a population at risk and should be targeted for health behavior interventions. Young Lithuanians have a relatively poor diet in terms of healthy eating following the standards of the Mediterranean diet. However, they meet recommendations to consume fruits, eat dishes with a homemade sauce, avoid overuse of butter, margarine or cream, drink low amounts of sweetened and carbonated beverages and avoid commercially baked sweets or pastries. The consumption of vegetables approaches the recommended norm. Better adherence to the Mediterranean diet among the Lithuanian young population is related to the female gender, higher physical activity, lower body mass index and higher education.

These study results should be considered by nutrition policymakers and public health authorities to improve policies and develop intervention plans for improving the nutrition habits of young adults in order to prevent health-damaging outcomes later in their lives.

## Figures and Tables

**Table 1:** Descriptive statistics and comparison of study variables between genders (total *n* = 3031).

Study Variable	Total *n* (%) or Mean ± SD	Male *n* (%) or Mean ± SD	Female *n* (%) or Mean ± SD
Mediterranean diet			
Poor	1043 (34.4)	776 (45)	267 (20.4) **
Average	1769 (58.4)	910 (52.8)	859 (65.7) **
Good	219 (7.2)	37 (2.1)	182 (13.9) **
BMI			
Not overweight	2266 (74.8)	1146 (66.6)	1120 (85.6)
Overweight or obese	762 (25.2)	574 (33.4) **	188 (14.4)
Age	23.72 ± 4.81	22.54 ± 2.58	25.26 ± 6.37 **
Education			
Lower than university/college	1824 (60.9)	1185 (68.8)	639 (50.3)
University/college	1170 (39.1)	538 (31.2)	632 (49.7) **
MVPA			
Not sufficient (<150 min/week)	974 (36.0)	597 (34.6)	378 (38.5)
Sufficient (≥150 min/week)	1731 (64.0)	1126 (65.4) *	605 (61.5)

Note: * significance of Student t or χ2 when * *p* < 0.05; ** *p* < 0.01; SD—Standard Deviation; BMI—body mass index; MVPA—moderate-to-vigorous physical activity.

**Table 2:** Predictors of better adherence to a Mediterranean diet among Lithuanian young adults aged 18–36 years old (hierarchical linear regression).

Variable	Standardized Beta		
	Model 1	Model 2	Model 3
Body mass index (overweight/obese)	−0.08 **	−0.05 *	−0.04 *
Gender (female)		0.26 **	0.26 **
Age		0.01	0.06 *
Education (higher)		0.30 **	0.23 **
MVPA (sufficient)			0.15 **
ΔR	0.005 **	0.19 **	0.02 **

Note: * *p* < 0.05; ** *p* < 0.01; MVPA—moderate-to-vigorous physical activity.

**Table 3:** Comparison of means and percentage distributions of eating habits among Lithuanian young adults aged 18–36 years old.

MEDAS and Food Items	All	Body Mass Index % or Mean ± SD		Gender % or Mean ± SD		Education % or Mean ± SD		MVPA % or Mean ± SD	
		Not Overweight	Overweight/Obese	Male	Female	University/College	Lower Than University/College	Sufficient	Not Sufficient
Total MEDAS score	6.35 ± 2.08	6.46 ± 2.1 **	6.02 ± 1.8	5.77 ± 1.7	7.10 ± 2.1 **	7.28 ± 2.0 **	5.79 ± 1.8	6.53 ± 1.9 **	5.66 ± 1.9
Olive oil as main culinary fat (yes)	19.3	18.9	20.3	14.1	28.2 **	28.7 **	14.0	18.9	20.3
Amount of olive oil/day (tbsp)	2.62 ± 2.6	2.63 ± 2.7	2.61 ± 2.5	2.25 ± 2.03	3.27 ± 3.45	3.00 ± 2.7 **	2.45 ± 2.6	2.81 ± 2.9 **	2.27 ± 2.2
Servings of vegetable/day (1 s = 200 g.)	1.75 ± 1.5	1.79 ± 1.7 *	1.64 ± 1.1	1.67 ± 1.2	1.88 ± 2.0 **	1.84 ± 1.2 *	1.70 ± 1.7	1.88 ± 1.1 **	1.54 ± 2.1
Fruit units/day	3.56 ± 2.8	3.61 ± 2.8	3.43 ± 2.7	3.61 ± 2.9	3.46 ± 2.7	3.42 ± 2.5	3.69 ± 3.0 *	3.90 ± 2.8 **	2.95 ± 2.7
Servings of red meat, hamburger, meat products/day (1 s = 100–150 g)	1.85 ± 1.2	1.82 ± 1.2	1.92 ± 1.3	2.08 ± 1.3 **	1.45 ± 0.8	1.63 ± 1.2	1.99 ± 1.2 **	1.94 ± 1.3 **	1.74 ± 1.0
Servings of butter, margarine, cream/day (1 s = 12 g)	0.96 ± 1.2	0.96 ± 1.3	0.94 ± 1.0	1.03 ± 1.3 **	0.82 ± 1.0	0.86 ± 1.1	1.01 ± 1.3 **	0.99 ± 1.3	0.92 ± 1.0
Sweet or carbonated beverages/day	0.47 ± 0.8	0.45 ± 0.8	0.52 ± 0.8 *	0.63 ± 0.9 **	0.19 ± 0.4	0.30 ± 0.6	0.58 ± 0.8 **	0.43 ± 0.7	0.57 ± 0.9 **
Glasses of wine/wk	0.46 ± 1.2	0.50 ± 1.2 **	0.33 ± 1.0	0.22 ± 0.9	0.78 ± 1.4 **	0.68 ± 1.4 **	0.31 ± 1.0	0.36 ± 1.1	0.40 ± 1.0
Servings of legumes/week (1 s = 150 g)	1.13 ± 1.4	1.17 ± 1.4 *	1.03 ± 1.3	1.12 ± 1.5	1.15 ± 1.1	1.44 ± 1.6 **	0.96 ± 1.2	1.32 ± 1.5 **	0.83 ± 1.1
Servings of fish or shellfish/wk (1 s = 150–200 g)	1.20 ± 1.3	1.19 ± 1.3	1.21 ± 1.4	1.14 ± 1.4	1.30 ± 1.2 **	1.41 ± 1.4 **	1.08 ± 1.3	1.29 ± 1.4 **	1.09 ± 1.2
Commercial sweets or pastries, times/wk	1.90 ± 1.8	1.89 ± 1.8	1.92 ± 1.8	1.90 ± 1.9	1.89 ± 1.6	1.76 ± 1.8	1.97 ± 1.9 **	1.85 ± 1.8	1.99 ± 1.9
Servings of nuts/week (1 s = 30 g)	1.98 ± 2.8	1.91 ± 2.8	2.16 ± 2.9 *	1.94 ± 3.2	2.06 ± 2.1	2.40 ± 2.7 **	1.76 ± 2.8	2.29 ± 3.2 **	1.45 ± 1.8
White meat instead of red or processed meat (yes)	69.0	70.3 *	65.9	63.7	78.3 **	76.5 **	64.7	70.7 **	64.5
Dishes seasoned with sofrito (times/wk)	3.08 ± 2.5	3.06 ± 2.5	3.13 ± 2.5	3.17 ± 2.7 **	2.91 ± 2.0	3.23 ± 2.5 *	2.99 ± 2.5	3.30 ± 2.7	2.73 ± 2.1

Note: significance of Student t or χ2: * *p* < 0.05; ** *p* < 0.01; MVPA—moderate-to-vigorous physical activity; MEDAS—Mediterranean Diet Adherence Screener; tbsp—tablespoon; s—servings; wk—week.

## Supplementary Materials

The following are available online at https://www.mdpi.com/2072-6643/12/7/2025/s1, Table S1: Comparison of means of eating habits between those who are not overweight and who are overweight/obese among Lithuanian young adults aged 18–36 years old, Table S2: Comparison of means of eating habits between genders among Lithuanian young adults aged 18–36 years old, Table S3: Comparison of means of eating habits between those with a university/college degree and those with less than a university/college degree among Lithuanian young adults aged 18–36 years old, Table S4: Comparison of means of eating habits between those who are sufficiently and not sufficiently physically active among Lithuanian young adults aged 18-36 years old.
