# Supplementary material for: Dietary Pattern and Its Correlates among Lithuanian Young Adults: Mediterranean Diet Approach
Source: Nutrients. 2020 Jul 8;12(7):2025. doi: 10.3390/nu12072025 (PMC7400829; doi:10.3390/nu12072025)
Supplement: Supplementary file 1 [file nutrients-12-02025-s001.zip › nutrients-842996-SI.docx]

Article

**Dietary pattern and its correlates among Lithuanian young adults: Mediterranean diet approach**

Brigita Mieziene^1*^, Arunas Emeljanovas^1^, Natalja Fatkulina^1^, Rimantas Stukas^1^

^1^ Institute of Health Sciences, Vilnius University, 03101 Vilnius, Lithuania, e-mail: [brigita](mailto:brigita.mieziene@mf.vu.lt)*[.](mailto:brigita.mieziene@mf.vu.lt)*[mieziene@mf.vu.lt](mailto:brigita.mieziene@mf.vu.lt);

^1^ Institute of Health Sciences, Vilnius University, 03101 Vilnius, Lithuania, e-mail: [arunas.emeljanovas@mf.vu.lt](mailto:arunas.emeljanovas@mf.vu.lt);

^1^ Institute of Health Sciences, Vilnius University, 03101 Vilnius, Lithuania, e-mail: [natalja.fatkulina@mf.vu.lt](mailto:natalja.fatkulina@mf.vu.lt);

^1^ Institute of Health Sciences, Vilnius University, 03101 Vilnius, Lithuania, e-mail: [rimantas.stukas@mf.vu.lt](mailto:rimantas.stukas@mf.vu.lt)

* Correspondence: brigita.mieziene@lsu.lt, Tel.: +370 68684622

Received: date; Accepted: date; Published: date

Table 1. Comparison of means of eating habits between those who are not overweight and who are overweight/obese among Lithuanian young adults aged 18–36 years old.

| **MEDAS and food items** | **Body mass index**  **Mean±SD** | | **Student t** | **Cohen d** |
| --- | --- | --- | --- | --- |
|  | Not overweight | Overweight/obese |  |  |
| Total MEDAS score | 6.46±2.1** | 6.02±1.8 | 5.069 | 0.22 |
| Amount of olive oil/day (tbsp) | 2.63±2.7 | 2.61±2.5 | 0.147 | 0.01 |
| Servings of vegetable /day (1s=200g.) | 1.79±1.7* | 1.64±1.1 | 2.188 | 0.10 |
| Fruit units/day | 3.61±2.8 | 3.43±2.7 | 1.427 | 0.06 |
| Servings of red meat, hamburger, meat products/day (1s=100-150g) | 1.82±1.2 | 1.92±1.3 | -1.896 | 0.08 |
| Servings of butter, margarine, cream/day (1s=12g) | 0.96±1.3 | 0.94±1.0 | 0.467 | 0.02 |
| Sweet or carbonated beverages/day | 0.45±0.8 | 0.52±0.8* | -2.253 | 0.08 |
| Glasses of wine/wk | 0.50±1.2** | 0.33±1.0 | 3.344 | 0.14 |
| Servings of legumes/week (1s=150g) | 1.17±1.4* | 1.03±1.3 | 2.317 | 0.10 |
| Servings of fish or shellfish/wk (1s=150-200g) | 1.19±1.3 | 1.21±1.4 | -0.337 | 0.01 |
| Commercial sweets or pastries, times/wk | 1.89±1.8 | 1.92±1.8 | -0.397 | 0.02 |
| Servings of nuts/week (1s=30g) | 1.91±2.8 | 2.16±2.9* | -1.983 | 0.35 |
| Dishes seasoned with sofrito (times/wk) | 3.06±2.5 | 3.13±2.5 | -0.609 | 0.03 |

Note: significance of Student t: *p < 0.05; ** p < 0.01; MVPA—moderate-to-vigorous physical activity; tbsp—tablespoon; s—servings; wk—week

Table 2. Comparison of means of eating habits between genders among Lithuanian young adults aged 18–36 years old.

| **MEDAS and Food items** | **Gender**  **Mean±SD** | | **Student t** | **Cohen d** |
| --- | --- | --- | --- | --- |
|  | Male | Female |  |  |
| Total MEDAS score | 5.77±1.7 | 7.10±2.1** | -18.412 | 0.69 |
| Amount of olive oil/day (tbsp) | 2.25±2.03 | 3.27±3.45 | -9.636 | 0.36 |
| Servings of vegetable /day (1s=200g.) | 1.67±1.2 | 1.88±2.0** | -3.387 | 0.12 |
| Fruit units/day | 3.61±2.9 | 3.46±2.7 | 1.352 | 0.05 |
| Servings of red meat, hamburger, meat products/day (1s=100-150g) | 2.08±1.3** | 1.45±0.8 | 12.993 | 0.55 |
| Servings of butter, margarine, cream/day (1s=12g) | 1.03±1.3** | 0.82±1.0 | 4.204 | 0.21 |
| Sweet or carbonated beverages/day | 0.63±0.9** | 0.19±0.4 | 13.492 | 0.59 |
| Glasses of wine/wk | 0.22±0.9 | 0.78±1.4** | -12.783 | 0.01 |
| Servings of legumes/week (1s=150g) | 1.12±1.5 | 1.15±1.1 | -0.503 | 0.02 |
| Servings of fish or shellfish/wk (1s=150-200g) | 1.14±1.4 | 1.30±1.2** | -2.920 | 0.12 |
| Commercial sweets or pastries, times/wk | 1.90±1.9 | 1.89±1.6 | 0.136 | 0.01 |
| Servings of nuts/week (1s=30g) | 1.94±3.2 | 2.06±2.1 | -1.050 | 0.04 |
| Dishes seasoned with sofrito (times/wk) | 3.17±2.7** | 2.91±2.0 | 2.582 | 0.10 |

Note: -significance of Student t: *p < 0.05; ** p < 0.01; MVPA—moderate-to-vigorous physical activity; tbsp—tablespoon; s—servings; wk—week

Table 3. Comparison of means of eating habits between those with a university/college degree and those with less than a university/college degree among Lithuanian young adults aged 18–36 years old.

| **MEDAS and Food items** | **Education**  **Mean±SD** | | **Student t** | **Cohen d** |
| --- | --- | --- | --- | --- |
|  | University/ college | Lower than university/ college |  |  |
| Total MEDAS score | 7.28±2.0** | 5.79±1.8 | -20.373 | 0.76 |
| Amount of olive oil/day (tbsp) | 3.00±2.7** | 2.45±2.6 | -5.155 | 0.20 |
| Servings of vegetable /day (1s=200g.) | 1.84±1.2* | 1.70±1.7 | -2.121 | 0.09 |
| Fruit units/day | 3.42±2.5 | 3.69±3.0* | 2.345 | 0.10 |
| Servings of red meat, hamburger, meat products/day (1s=100-150g) | 1.63±1.2 | 1.99±1.2** | 7.235 | 0.29 |
| Servings of butter, margarine, cream/day (1s=12g) | 0.86±1.1 | 1.01±1.3** | 2.832 | 0.12 |
| Sweet or carbonated beverages/day | 0.30±0.6 | 0.58±0.8** | 8.619 | 0.35 |
| Glasses of wine/wk | 0.68±1.4** | 0.31±1.0 | -8.201 | 0.29 |
| Servings of legumes/week (1s=150g) | 1.44±1.6** | 0.96±1.2 | -8.646 | 0.33 |
| Servings of fish or shellfish/wk (1s=150-200g) | 1.41±1.4** | 1.08±1.3 | -5.936 | 0.23 |
| Commercial sweets or pastries, times/wk | 1.76±1.8 | 1.97±1.9** | 2.715 | 0.11 |
| Servings of nuts/week (1s=30g) | 2.40±2.7** | 1.76±2.8 | -5.595 | 0.23 |
| Dishes seasoned with sofrito (times/wk) | 3.23±2.5* | 2.99±2.5 | -2.300 | 0.09 |

Note: significance of Student t: *p < 0.05; ** p < 0.01; MVPA—moderate-to-vigorous physical activity; tbsp—tablespoon; s—servings; wk—week

Table 4. Comparison of means of eating habits between those who are sufficiently and not sufficiently physically active among Lithuanian young adults aged 18-36 years old.

| **MEDAS and Food items** | **MVPA**  **Mean±SD** | | **Student t** | **Cohen d** |
| --- | --- | --- | --- | --- |
|  | Sufficient | Not sufficient |  |  |
| Total MEDAS score | 6.53±1.9** | 5.66±1.9 | -11.377 | 0.45 |
| Amount of olive oil/day (tbsp) | 2.81±2.9** | 2.27±2.2 | -4.912 | 0.21 |
| Servings of vegetable /day (1s=200g.) | 1.88±1.1** | 1.54±2.1 | -5.199 | 0.17 |
| Fruit units/day | 3.90±2.8** | 2.95±2.7 | -8.153 | 0.33 |
| Servings of red meat, hamburger, meat products/day (1s=100-150g) | 1.94±1.3** | 1.74±1.0 | -3.723 | 0.16 |
| Servings of butter, margarine, cream/day (1s=12g) | 0.99±1.3 | 0.92±1.0 | -1.171 | 0.06 |
| Sweet or carbonated beverages/day | 0.43±0.7 | 0.57±0.9** | 4.071 | 0.25 |
| Glasses of wine/wk | 0.36±1.1 | 0.40±1.0 | 0.904 | 0.04 |
| Servings of legumes/week (1s=150g) | 1.32±1.5** | 0.83±1.1 | -8.567 | 0.36 |
| Servings of fish or shellfish/wk (1s=150-200g) | 1.29±1.4** | 1.09±1.2 | -3.559 | 0.15 |
| Commercial sweets or pastries, times/wk | 1.85±1.8 | 1.99±1.9 | 1.851 | 0.07 |
| Servings of nuts/week (1s=30g) | 2.29±3.2** | 1.45±1.8 | -7.201 | 0.31 |
| Dishes seasoned with sofrito (times/wk) | 3.30±2.7 | 2.73±2.1 | -5.545 | 0.23 |

Note: significance of Student t: *p < 0.05; ** p < 0.01; MVPA—moderate-to-vigorous physical activity; tbsp—tablespoon; s—servings; wk—week
